# Age- and sex-specific hospital bed-day rates in people with and without type 2 diabetes: A territory-wide population-based cohort study of 1.5 million people in Hong Kong

**DOI:** 10.1371/journal.pmed.1004261

**Published:** 2023-08-04

**Authors:** Hongjiang Wu, Aimin Yang, Eric S. H. Lau, Xinge Zhang, Baoqi Fan, Mai Shi, Chuiguo Huang, Ronald C. W. Ma, Alice P. S. Kong, Elaine Chow, Wing-Yee So, Juliana C. N. Chan, Andrea O. Y. Luk

**Affiliations:** 1 Department of Medicine and Therapeutics, The Chinese University of Hong Kong, Hong Kong Special Administrative Region, People’s Republic of China; 2 Hong Kong Institute of Diabetes and Obesity, The Chinese University of Hong Kong, Hong Kong Special Administrative Region, People’s Republic of China; 3 Li Ka Shing Institute of Health Sciences, The Chinese University of Hong Kong, Hong Kong Special Administrative Region, People’s Republic of China; 4 Hong Kong Hospital Authority, Hong Kong Special Administrative Region, People’s Republic of China

## Abstract

**Background:**

Type 2 diabetes affects multiple systems. We aimed to compare age- and sex-specific rates of all-cause and cause-specific hospital bed-days between people with and without type 2 diabetes.

**Methods and findings:**

Data were provided by the Hong Kong Hospital Authority. We included 1,516,508 one-to-one matched people with incident type 2 diabetes (*n* = 758,254) and those without diabetes during the entire follow-up period (*n* = 758,254) between 2002 and 2018, followed until 2019. People with type 2 diabetes and controls were matched for age at index date (±2 years), sex, and index year (±2 years). We defined hospital bed-day rate as total inpatient bed-days divided by follow-up time. We constructed negative binominal regression models to estimate hospital bed-day rate ratios (RRs) by age at diabetes diagnosis and sex. All RRs were stratified by sex and adjusted for age and index year. During a median of 7.8 years of follow-up, 60.5% (*n* = 459,440) of people with type 2 diabetes and 56.5% (*n* = 428,296) of controls had a hospital admission for any cause, with a hospital bed-day rate of 3,359 bed-days and 2,350 bed-days per 1,000 person-years, respectively. All-cause hospital bed-day rate increased with increasing age in controls, but showed a J-shaped relationship with age in people with type 2 diabetes, with 38.4% of bed-days in those diagnosed <40 years caused by mental health disorders. Type 2 diabetes was associated with increased risks for a wide range of medical conditions, with an RR of 1.75 (95% CI [confidence interval] [1.73, 1.76]; *p* < 0.001) for all-cause hospital bed-days in men and 1.87 (95% CI [1.85, 1.89]; *p* < 0.001) in women. The RRs were greater in people with diabetes diagnosed at a younger than older age and varied by sex according to medical conditions. Sex differences were most notable for a higher RR for urinary tract infection and peptic ulcer, and a lower RR for chronic kidney disease and pancreatic disease in women than men. The main limitation of the study was that young people without diabetes in the database were unlikely to be representative of those in the Hong Kong general population with potential selection bias due to inclusion of individuals in need of medical care.

**Conclusions:**

In this study, we observed that type 2 diabetes was associated with increased risks of hospital bed-days for a wide range of medical conditions, with an excess burden of mental health disorders in people diagnosed at a young age. Age and sex differences should be considered in planning preventive and therapeutic strategies for type 2 diabetes. Effective control of risk factors with a focus on mental health disorders are urgently needed in young people with type 2 diabetes. Healthcare systems and policymakers should consider allocating adequate resources and developing strategies to meet the mental health needs of young people with type 2 diabetes, including integrating mental health services into diabetes care.

## Abstract 2

In an observational cohort study, Hongjiang Wu and colleagues analyse data from more than 1.5 million people Hong Kong between 2002 and 2018 and compare age- and sex-specific rates of all-cause and cause-specific hospital bed-day rates in those with and without type 2 diabetes.

## Author summary

**Why was this study done?:**

- Previous studies examining the association between type 2 diabetes and medical conditions focused on the risk of time-to-first event, which does not capture an individual’s disease burden, as many medical conditions can lead to multiple hospital admissions.
- Hospital bed-day rate, defined as cumulative inpatient bed-days per person-time, is a health metric that provides direct estimates on risk of total bed-days associated with a medical condition and indirectly reflects disease severity and inpatient resource use.
- The association between type 2 diabetes and all-cause and cause-specific hospital bed-day rates remains unclear.

**What did the researchers do and find?:**

- We analysed data from 758,254 people with incident type 2 diabetes and 758,254 matched controls without diabetes in Hong Kong between 2002 and 2018. We compared age- and sex-specific rates of all-cause and cause-specific hospital bed-day rates between the 2 groups.
- All-cause hospital bed-day rate increased with increasing age in controls, but showed a J-shaped relationship with age in people with type 2 diabetes. Among people with type 2 diabetes diagnosed <40 years, 38.4% of hospital bed-days were caused by mental health disorders.
- Type 2 diabetes was associated with increased risks of hospital bed-day rate for a wide range of medical conditions, with the rate ratios (RRs) greater in people with diabetes diagnosed at a younger than older age and varied by sex according to medical conditions. Compared to men, women had a significantly higher RR for urinary tract infection and peptic ulcer but a lower RR for chronic kidney disease and pancreatic disease.

**What do these findings mean?:**

- It is crucial to consider age and sex differences when planning preventive and therapeutic strategies for type 2 diabetes to reduce hospital bed-days.
- Efforts to provide integrated care and effectively manage risk factors, with a focus on mental health disorders, are urgently needed in young people with type 2 diabetes.

## Introduction

Type 2 diabetes is a complex multisystem disorder that affects about 1 in 10 adults worldwide [1]. The global epidemiology of type 2 diabetes is changing, characterised by a trend towards younger age at diagnosis [2,3] and an increase in the burden of newly emerging complications, such as cancer and liver disease [4], although cardiovascular and renal complications are still the leading causes of morbidity in people with type 2 diabetes.

Studies of the association between type 2 diabetes and medical conditions traditionally focused on the risk of time-to-first event and were restricted to a small set of health outcomes. However, time-to-first event fails to capture an individual’s disease burden, as many medical conditions are nonfatal recurrent events that may occur repeatedly leading to multiple hospital admissions. Hospital bed-day rate, defined as cumulative inpatient bed-days per person-time [5], is an alternative health metric that provides direct estimates on risk of total bed-days associated with a medical condition and indirectly reflects disease severity and inpatient resource use. It provides more comprehensive information about the disease progression and additional insights about the burden on healthcare system. To our knowledge, no studies have examined the association between type 2 diabetes and hospital bed-day rate in a comprehensive manner.

Using data from the Hong Kong Diabetes Register that enrolled patients with diabetes attending hospital-based clinics, we have reported a higher risk of hospital bed-day rate for major diabetes-related complications in people with young-onset (diagnosis age <40 years) than usual-onset (diagnosis age ≥40 years) type 2 diabetes [5]. However, due to a lack of a comparison group without diabetes, it remains unclear whether the increased risk of hospital bed-day in young-onset type 2 diabetes is independent of longer disease duration and whether there are differences in patterns of hospital bed-days between people with and without diabetes.

In this study, we extended our previous research [5] from traditional complications of type 2 diabetes to a broad range of medical conditions and from a hospital-clinic-based diabetes register to the entire population with incident type 2 diabetes and matched controls without diabetes in Hong Kong. We hypothesised that type 2 diabetes is associated with an increased risk of hospital bed-days for various medical conditions. We aimed to provide a comprehensive analysis to compare age- and sex-specific rates of all-cause and cause-specific hospital bed-days between people with and without type 2 diabetes.

## Methods

### Ethics statement

Approval of the study was obtained from the Chinese University of Hong Kong-New Territories East Cluster Clinical Research Ethics Committee (CREC Ref. No. 2020.032). The requirement for individual patient consent was waived as this study was based on anonymised data from electronic medical record (EMR).

### Study setting and data source

Hong Kong is a special administrative region of China with a population of about 7.5 million. The Hong Kong Hospital Authority (HA) is a statutory body that governs all hospitals and majority of out-patient clinics in the public sector, providing about 90% of all healthcare services in Hong Kong. In 2000, the Hong Kong HA adopted a territory-wide EMR system to routinely collect clinical information for all people attending public hospitals and clinics. We obtained data of all people who have ever had a glucose measurement in the HA EMR system, comprising approximately 60% of people living in Hong Kong. The HA database is broadly representative of the Hong Kong general population in terms of all-cause hospitalisation rate and mortality rate [6,7]. Detailed information on the database has been previously reported [7–9]. We planned the study in May 2022, conducted the analyses between June and December 2022, and performed additional analyses in April 2023 in response to suggestions of journal reviewers. This study is reported as per the Strengthening the Reporting of Observational Studies in Epidemiology (STROBE) guideline (S1 Checklist).

### Study population

We included all people (*n* = 758,254) with incident (new-onset) type 2 diabetes between 1 January 2002 and 31 December 2018 and aged 18 to 99 years at diabetes diagnosis in the database. A wash-out period of 2 years from 2000 to 2001 was used to exclude people with preexisting diabetes. Incident type 2 diabetes was based on physician diagnosis, laboratory results, and prescriptions of medications [10]. For each individual with incident type 2 diabetes, we randomly selected 1 control who did not have diabetes during the entire follow-up period, matched for age at index date (±2 years), sex, and index year (±2 years) (**S1 Fig**). Matching was performed without replacement and a 2-year range matching criterion for age and index year is a reasonable balance between achieving adequate matching quality and maintaining a sufficient sample size. We defined index date for people with type 2 diabetes as the date of diabetes diagnosis. For controls, we randomly assigned an index date to each of them before matching process based on the distribution of index dates in people with type 2 diabetes. Women with gestational diabetes were excluded from the control group. People with preexisting medical conditions at the index date were not excluded from the analyses because we aimed to compare the burden of hospital bed-days in people with and without type 2 diabetes, rather than examining the independent association between diabetes and risk of the first hospitalisation event.

### Follow-up time and outcomes

We followed the study population from index date to 31 December 2019 or date of death, whichever came first. The outcomes of this study were all-cause and cause-specific hospital bed-day rates. We defined hospital bed-day rate as total number of inpatient bed-days divided by follow-up time in person-years [5]. We obtained all hospital admission records from the HA EMR, which were coded using the International Classification of Diseases, Ninth Revision (ICD-9). We excluded: (1) day-case admissions (defined as admission and discharge on the same day), which were mainly for assessment, review, and/or noninvasive intervention; (2) admissions with an extremely long stay (≥365 days in a single admission), which were likely long-term convalescent placements; and (3) pregnancy/delivery-related admissions (ICD-9: 630–679) from the analyses. We used principal diagnosis codes at discharge to classify the causes of hospitalisation. We calculated hospital bed-day rates for all-cause and for 7 broad disease categories as follows: infection/parasites (ICD-9: 001–139), neoplasms (ICD-9: 140–239), mental health disorders (ICD-9: 290–319), circulatory system (ICD-9: 390–459), respiratory system (ICD-9: 460–519), digestive system (ICD-9: 520–579), and genitourinary system (ICD-9: 580–629). These disease categories include both traditional complications of diabetes with well-established causal relationships (e.g., cardiovascular diseases and kidney diseases) and emerging complications of diabetes with increasing evidence of a higher risk of them in people with diabetes (e.g., cancers and liver disease). In addition, we assessed 72 (68 for men and 70 for women) selected specific medical conditions, which contributed to >1% of inpatient bed-days among each broad disease category (**S1 Table**).

### Statistical analysis

We performed all analyses separately for men and women and by age group at index date (18 to 39, 40 to 59, 60 to 79, and 80 to 99 years). Hospital bed-day rate was expressed as number of bed-days per 1,000 person-years. Given the overdispersion of the data, we constructed negative binominal regression models to estimate hospital bed-day rate ratios (RRs) with 95% confidence intervals (CIs) comparing people with and without type 2 diabetes. All models were adjusted for age and index year, although there was little difference in RRs between unadjusted and adjusted models as a consequence of using matching design [11]. To provide more comprehensive information to inform public health interventions, we also calculated the excess absolute risk of hospital bed-days associated with type 2 diabetes, which represents the difference in the rate of hospital bed-days between people with type 2 diabetes and controls. A two-tailed *p*-value less than 0.05 was considered statistically significant in the primary analyses. Given that our analyses included a large number of outcomes, we additionally corrected for *p*-values for multiple comparisons using the Bonferroni method to reduce the probability of false-positive results. A *p*-value less than 0.0036 (= 0.05/14 broad disease categories in 2 sexes) for broad disease categories and a *p*-value less than 0.00036 (= 0.05/138 medical conditions in 2 sexes) for selected medical conditions in two-tailed tests were considered statistically significant after adjustment for multiple comparisons.

We performed all analyses using R software, version 4.0.3 (R Foundation for Statistical Computing, Vienna, Austria).

## Results

A total number of 1,516,508 one-to-one matched people with incident type 2 diabetes and controls without diabetes were included in the analysis. Among people with type 2 diabetes, 51.6% of them were men, the mean (standard deviation) age at diabetes diagnosis was 62.8 (13.1) years, and 4.1% were diagnosed under the age of 40 years (**S2 Table**). Men were diagnosed with type 2 diabetes roughly 2 years younger than women (61.9 [12.7] versus 63.8 [13.5] years).

### Absolute risk of hospital bed-days

During a median (interquartile range) of 7.8 (4 to 12) years of follow-up (with 12.5 million person-years), 60.5% (*n* = 459,440) of people with type 2 diabetes and 56.5% (*n* = 428,296) of controls had been admitted to hospital for any cause, with a crude all-cause hospital bed-day rate of 3,359 bed-days and 2,350 bed-days per 1,000 person-years, respectively (**Table 1**). Among the 7 broad disease categories, circulatory conditions and respiratory conditions contributed to the largest number of inpatient bed-days in both people with type 2 diabetes and controls. The number of people with hospitalisations for each single broad disease category and their combinations is shown in **S2 Fig**. The crude hospital admission rate and median inpatient bed-days for each admission are shown in **S3 Table**.

**Table 1:** Inpatient bed-days and crude hospital bed-day rates for broad disease categories in people with and without type 2 diabetes.

Causes of hospitalisation	Type 2 diabetes (*N* = 758,254)			Controls (*N* = 758,254)			
	Number of people with hospitalisation (% of all people)	Inpatient bed-days (% of all bed-days)	Crude hospital bed-day rate (per 1,000 person-years)	Number of people with hospitalisation (% of all people)	Inpatient bed-days (% of all bed-days)	Crude hospital bed-day rate (per 1,000 person-years)	Adjusted hospital bed-day rate ratio*
**Men (*N* = 783,000)**							
Infection/parasites	29,818 (7.6)	464,283 (4.4)	153.1	21,270 (5.4)	323,368 (4.1)	101.8	2.11 (2.04, 2.18)
Neoplasms	42,011 (10.7)	1,132,124 (10.6)	373.4	40,998 (10.5)	1,120,542 (14.1)	352.9	1.53 (1.49, 1.58)
Mental health disorders	12,591 (3.2)	615,451 (5.8)	203.0	11,823 (3.0)	524,575 (6.6)	165.2	1.13 (1.07, 1.20)
Circulatory system	89,295 (22.8)	1,799,644 (16.9)	593.5	62,880 (16.1)	1,054,251 (13.3)	332.0	2.27 (2.23, 2.32)
Respiratory system	63,633 (16.3)	1,557,922 (14.6)	513.8	59,680 (15.2)	1,555,735 (19.6)	489.9	1.46 (1.43, 1.49)
Digestive system	66,184 (16.9)	795,691 (7.5)	262.4	58,745 (15.0)	601,639 (7.6)	189.5	1.68 (1.65, 1.72)
Genitourinary system	48,027 (12.3)	724,963 (6.8)	239.1	34,213 (8.7)	369,695 (4.7)	116.4	2.94 (2.86, 3.01)
All-cause	239,150 (61.1)	10,669,349 (100)	3,518.9	226,170 (57.8)	7,920,791 (100)	2,494.5	1.75 (1.73, 1.76)
**Women (*N* = 733,508)**							
Infection/parasites	29,179 (8.0)	364,202 (3.7)	117.7	20,258 (5.5)	236,347 (3.3)	73.7	2.30 (2.23, 2.38)
Neoplasms	35,901 (9.8)	885,143 (8.9)	286.0	31,819 (8.7)	762,860 (10.8)	238.0	1.68 (1.63, 1.74)
Mental health disorders	14,874 (4.1)	689,070 (7.0)	222.7	13,275 (3.6)	485,877 (6.9)	151.6	1.39 (1.31, 1.46)
Circulatory system	76,204 (20.8)	1,697,764 (17.1)	548.6	56,033 (15.3)	1,036,210 (14.7)	323.3	2.25 (2.20, 2.30)
Respiratory system	52,839 (14.4)	992,821 (10.0)	320.8	47,092 (12.8)	907,262 (12.8)	283.1	1.67 (1.63, 1.71)
Digestive system	55,007 (15.0)	658,013 (6.6)	212.6	45,392 (12.4)	485,805 (6.9)	151.6	1.76 (1.72, 1.80)
Genitourinary system	49,606 (13.5)	733,492 (7.4)	237.0	31,986 (8.7)	358,699 (5.1)	111.9	2.64 (2.57, 2.71)
All-cause	220,290 (60.1)	9,913,789 (100)	3,203.6	202,126 (55.1)	7,069,885 (100)	2,205.9	1.87 (1.85, 1.89)

* Based on negative binomial regression models with adjustment for age and index year.

**Fig 1:**
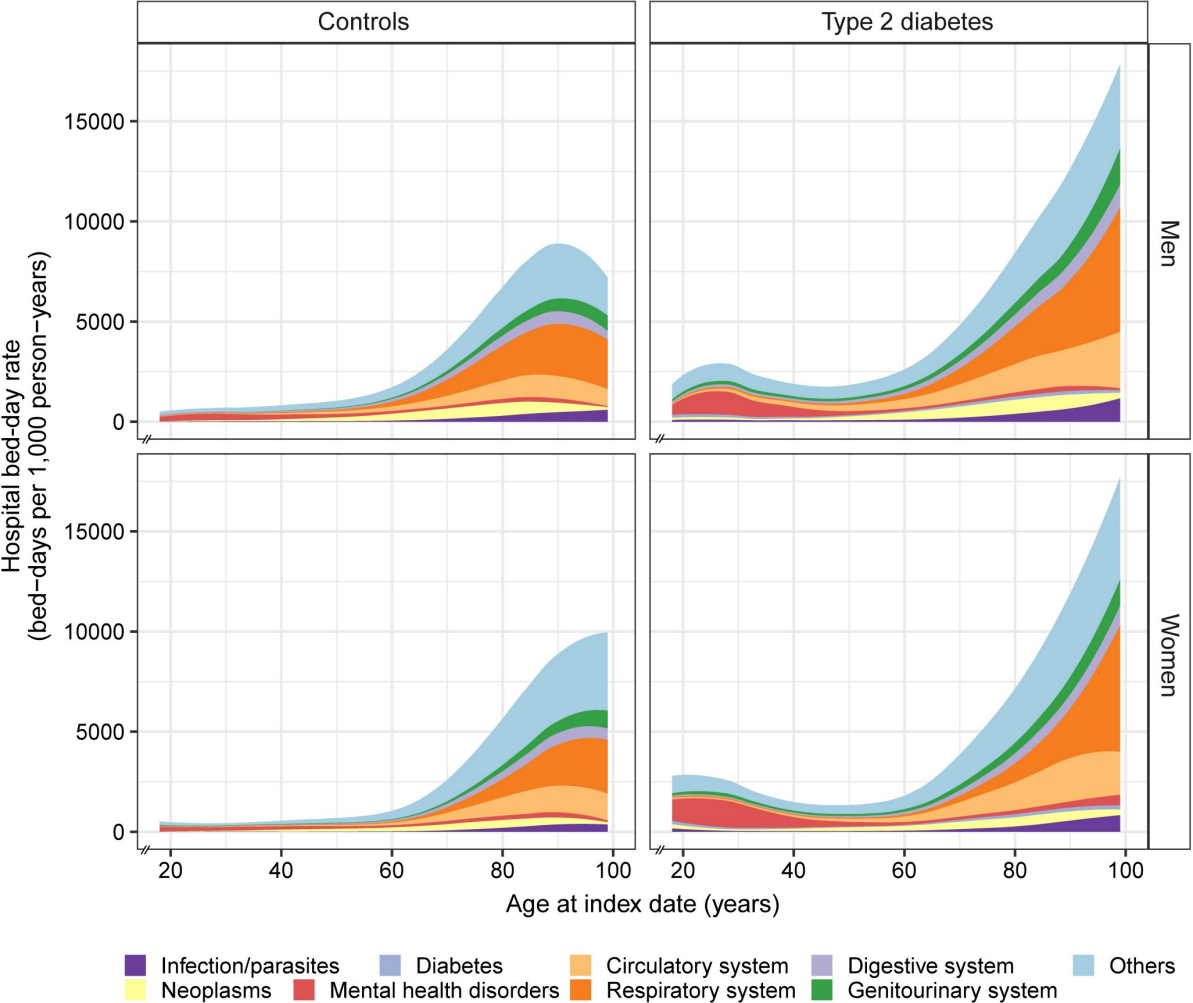
Crude hospital bed-day rates for broad disease categories by age at index date in people with and without type 2 diabetes. The x-axis begins at 18 years at index date.

**Fig 2:**
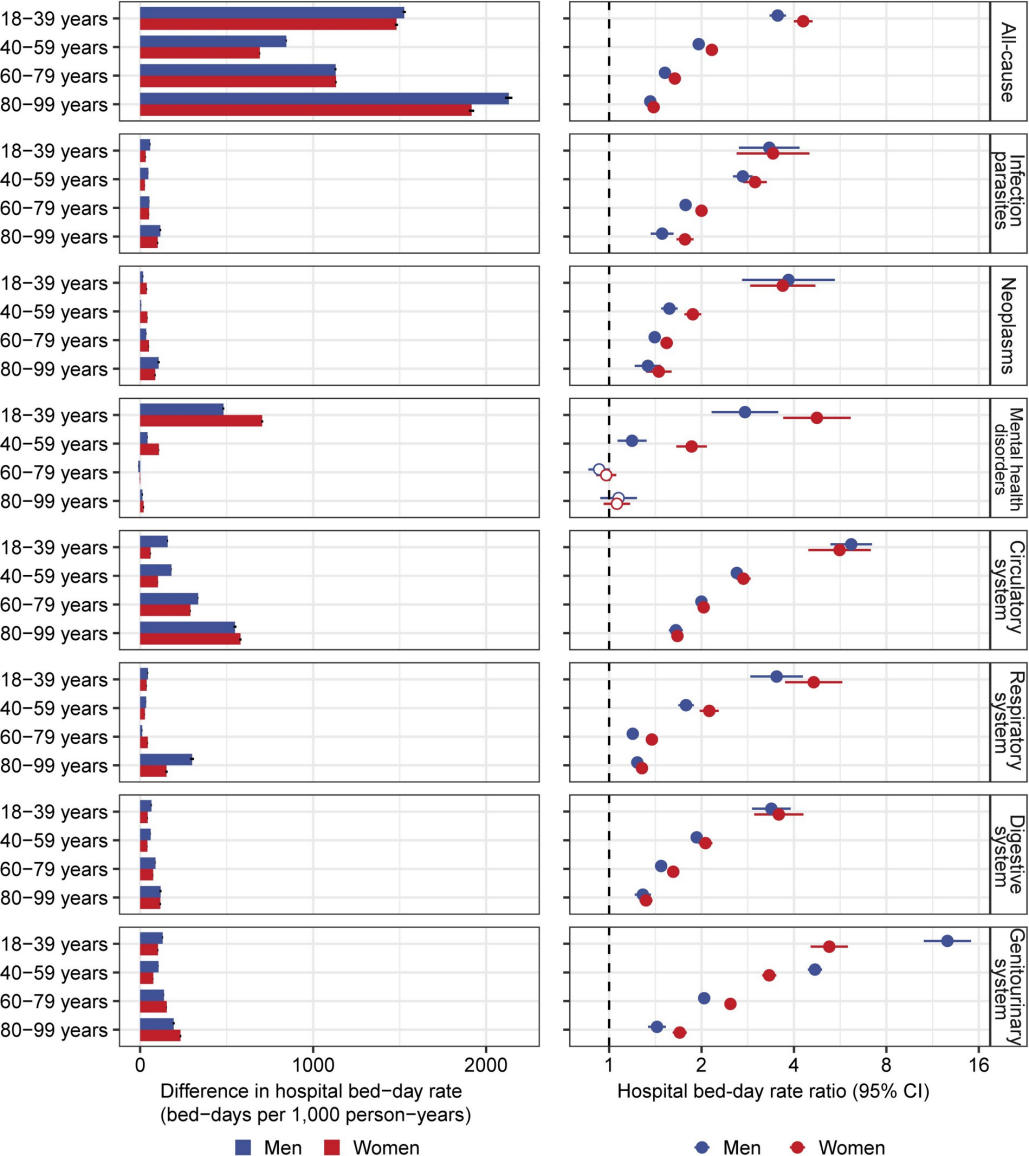
Age- and sex-specific excess absolute risk of hospital bed-days and hospital bed-day RRs for broad disease categories comparing people with and without type 2 diabetes. All hospital bed-day RRs were adjusted for age and index year. Dots are point estimates and lines are 95% CIs. CI, confidence interval; RR, rate ratio.

The age-specific patterns of hospital bed-day rate differed substantially between people with type 2 diabetes and controls (**Fig 1**). In people with type 2 diabetes, there was a J-shaped relationship between age at diabetes diagnosis and rate of all-cause hospital bed-days, with the rate higher in people diagnosed at <40 years and ≥60 years than those diagnosed at 40 to 59 years. In controls, the rate of all-cause hospital bed-days increased constantly with age. In people with type 2 diabetes diagnosed under the age of 40 years, mental health disorders were the leading cause of hospital bed-days, accounting for 38.4% of all bed-days, with the proportion being significantly higher in women (45.4%) than men (33.0%) (**Fig 1**). With increasing age at diabetes diagnosis, the predominant causes of hospital bed-days shifted from mental health disorders to circulatory conditions and respiratory conditions. Hospital bed-day rates for all the broad disease categories were higher in people with type 2 diabetes than controls (**Fig 2** and **Table 1**). The excess absolute risk of hospital bed-days for the broad disease categories associated with type 2 diabetes was generally greater in older than younger people, except for mental health disorders. The excess absolute risk of hospital bed-day rates for mental health disorders was greater in younger as compared to older people, particularly in women (**Fig 2**) and largely due to schizophrenia and bipolar disorder (**S3 Fig**).

Among the 72 selected medical conditions, the highest hospital bed-day rates were observed for pneumonia, ischemic stroke, chronic obstructive pulmonary disease, heart failure, and ischemic heart disease in both people with type 2 diabetes and controls (**S4 Fig**). Men had higher absolute rates of hospital bed-days for all-cause and for the majority of medical conditions, with the exception of urinary tract infection, heart failure, and certain mental health disorders being higher in women than men.

### Relative risk of hospital bed-days comparing people with type 2 diabetes and controls

Compared to controls, type 2 diabetes was associated with 1.75-fold (95% CI [1.73, 1.76]; *p* < 0.001) and 1.87-fold (95% CI [1.85, 1.89]; *p* < 0.001) increased risk of all-cause hospital bed-days in men and women, respectively (**Table 1**). The RRs for the broad disease categories were generally greater in women than men, except for genitourinary system diagnoses. In both sexes, the highest RR was observed for genitourinary system diagnoses (RR in men: 2.94, 95% CI [2.86, 3.01]; *p* < 0.001; RR in women: 2.64, 95% CI [2.57, 2.71]; *p* < 0.001) and the lowest for mental health disorders (RR in men: 1.13, 95% CI [1.07, 1.20]; *p* < 0.001; RR in women: 1.39, 95% CI [1.31, 1.46]; *p* < 0.001). The RRs for all the broad disease categories remained statistically significant in both sexes after Bonferroni correction.

The RRs for the association between type 2 diabetes and hospital bed-day rates were significantly higher in people with younger than older age at diabetes diagnosis in both sexes (**Fig 2** and **S4 Table**). The RR for all-cause hospital bed-days was 3.54 (95% CI [3.33, 3.76]; *p* < 0.001) in men and 4.29 (95% CI [4.00, 4.60]; *p* < 0.001) in women with type 2 diabetes diagnosed at 18 to 39 years and decreased to 1.36 (95% CI [1.33, 1.40]; *p* < 0.001) in men and 1.40 (95% CI [1.37, 1.42]; *p* < 0.001) in women diagnosed at 80 to 99 years. Sex differences in RR varied by age and were most evident for mental health disorders and genitourinary conditions (**Fig 2**). The increased risk of hospital bed-days for mental health disorders in people with type 2 diabetes was only observed for those aged <60 years at index date, with the association consistently stronger in women than men. In contrast, RR for genitourinary system was greater in men than women at ages of <60 years, but reversed between sexes in older ages.

**Fig 3:**
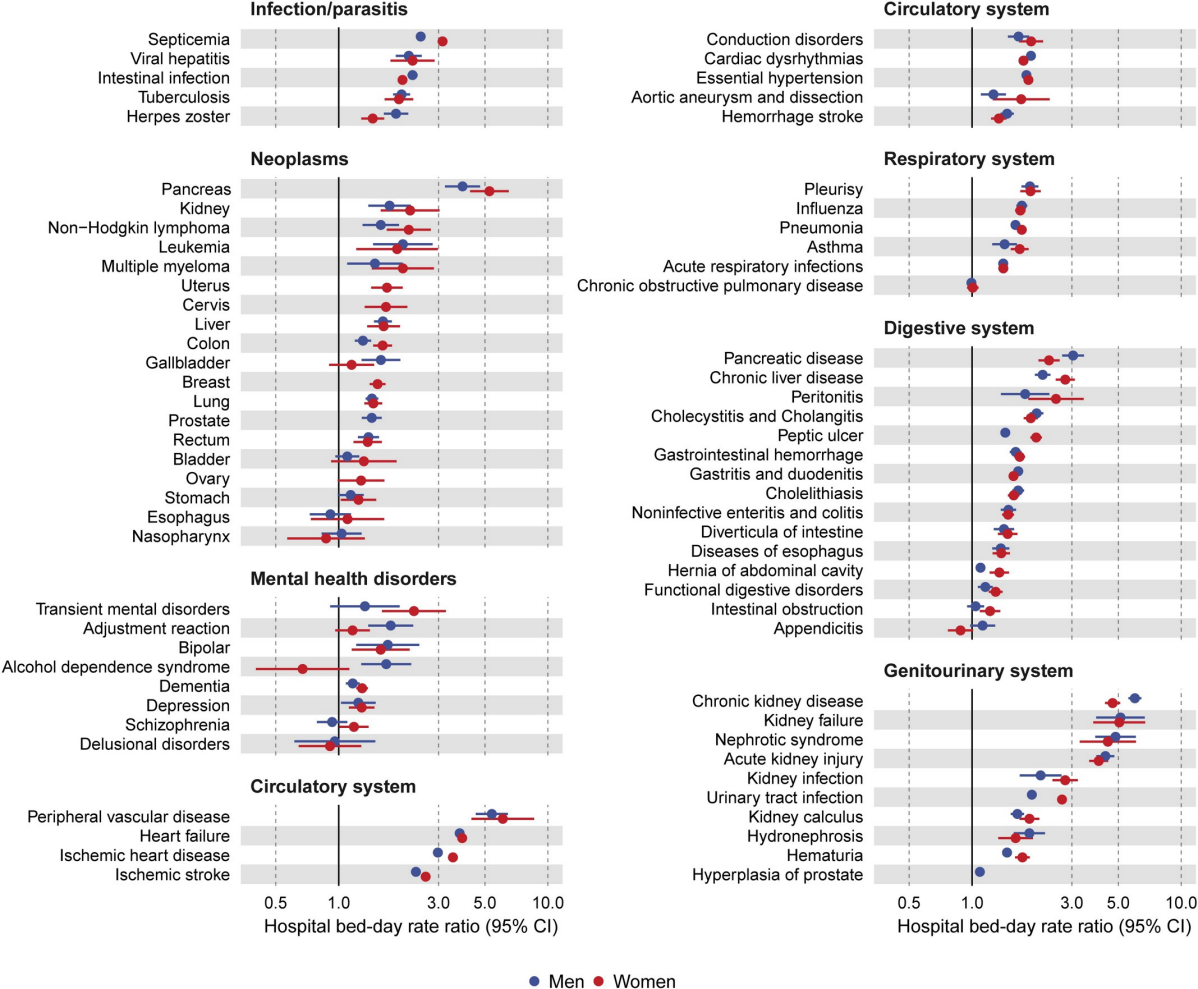
Sex-specific hospital bed-day RRs for the selected medical conditions comparing people with and without type 2 diabetes. All hospital bed-day RRs were adjusted for age and index year. Dots are point estimates and lines are 95% CIs. A filled circle indicates that the association remained statistically significant after Bonferroni correction (*p*-value less than 0.00036). CI, confidence interval; RR, rate ratio.

Among the 72 selected medical conditions, 58 of them in men and 60 in women had an RR greater than 1 and was statistically significant at *p*-value of 0.05 (**Fig 3** and **S5 Table**). After Bonferroni correction, the RRs for 53 medical conditions in both sexes remained statistically significant (**Fig 3** and **S6 Table**). The greatest RR was 6.00 (95% CI [5.59, 6.45]; *p* < 0.001) for chronic kidney disease and 5.39 (95% CI [4.52, 6.44]; *p* < 0.001) for peripheral vascular disease in men, and 6.09 (95% CI [4.31, 8.59]; *p* < 0.001) for peripheral vascular disease and 5.25 (95% CI [4.25, 6.50]; *p* < 0.001) for pancreatic cancer in women. Similarly, the RRs for most medical conditions were greatest in the youngest age group and declined with increasing age at diabetes diagnosis in both sexes (**S5 Fig**). Sex differences were most notable for a higher RR for urinary tract infection, peptic ulcer, septicemia, and ischemic heart disease and a lower RR for chronic kidney disease and pancreatic disease in women than men (**Fig 3**).

## Discussion

In this territory-wide population-based cohort study of over 1.5 million people, we provide a comprehensive landscape on the burden of hospital bed-days for 7 broad disease categories and 72 selected medical conditions in people with versus those without type 2 diabetes with 3 key findings. First, presence of type 2 diabetes was associated with increased risks of hospital bed-days for a broad range of medical conditions. Second, absolute risk of all-cause hospital bed-days increased with increasing age in people without diabetes, but showed a J-shaped relationship with age in people with type 2 diabetes characterised by a considerably high burden of bed-days due to mental health disorders in young-onset type 2 diabetes. Third, there were important age- and sex-related disparities in relative risk of hospital bed-days associated with type 2 diabetes, with the relative risks being substantially greater in people diagnosed at a younger than older age and varying between the sexes by different medical conditions.

Our study provided novel estimates and new insights into the burden of hospital bed-days in people with and without type 2 diabetes. The magnitude of relative risk for hospital bed-days associated with type 2 diabetes was greater than that reported for time-to-first hospitalisation event in the previous studies. For example, previous studies have reported that type 2 diabetes was associated with a 2-fold increased risk of first incident heart failure [12] and pancreatic cancer [13]. In our study, we observed a 4- to 5-fold increased risk of inpatient-bed days for heart failure and pancreatic cancer in people with type 2 diabetes as compared to controls. The discrepancy may be attributable to the choice of outcome measure, as hospital bed-days take into account factors leading to hospitalisation as well as those affecting the length of stay. Importantly, the association between type 2 diabetes and hospital bed-days extended to a broad range of medical conditions that had not been conventionally attributed to diabetes such as diseases of the respiratory and digestive systems. This finding was supported by an Australian study, which observed that people with type 2 diabetes had an increased hospitalisation rate for most medical conditions at ICD-10 three-digit diagnosis level compared to the general population [14]. The Australian study found that infections (e.g., gastrointestinal infections and respiratory infections) and mental health disorders (e.g., schizophrenia and depression) were emerging as leading causes of hospitalisation in people with diabetes and their excess hospitalisations associated with type 2 diabetes exceeded that of many traditional complications. The increased burden of nontraditional complications in diabetes was also reported by an analysis of UK primary care data, which found that there was a shift in leading causes of hospitalisation in people with diabetes from vascular diseases to nontraditional complications, such as liver disease and respiratory infections [15].

The explanations for the high hospital bed-day rates in people with type 2 diabetes are multifactorial and may include both causal and noncausal mechanisms. Type 2 diabetes may causally increase the risk of first and recurrent medical conditions requiring hospitalisations such as cardiovascular and kidney diseases. Through shared social, behavioural, environmental, and genetic risk factors, type 2 diabetes may also be indirectly linked to development or exacerbation of other medical conditions such as mental health disorders [16–18]. Lastly, the frequent coexistence of multiple cardiometabolic risk factors, other morbidities, and polypharmacy in people with diabetes increase their vulnerabilities during each hospitalisation, potentially contribute to more serious manifestation of the presenting illness and reduced response to treatment, thus prolong the length of hospital stay [19,20].

The very large sample size has allowed us to examine detailed age- and sex-related differences in hospital bed-day rates for a large number of medical conditions. We observed that the relative risk of hospital bed-days was most pronounced in the youngest age group and declined with increasing age at diabetes diagnosis. By studying a cohort of incident type 2 diabetes and including a comparison group without diabetes, we were able to disentangle the impact of age at diabetes diagnosis independent of diabetes duration and demonstrate the inherently aggressive nature of type 2 diabetes in young age [21,22]. In addition to clinical inertia and insufficient management guidelines for young-onset type 2 diabetes [23], people with type 2 diabetes diagnosed at a younger age have more rapid deterioration of β-cell function [24], worse metabolic risk profiles [25], and poorer response to glucose-lowering treatment [26] than those diagnosed at an older age. These factors, together with long disease duration, may result in a particularly high lifetime burden of morbidity in young-onset type 2 diabetes.

The J-shaped association of age with hospital bed-day rates in people with type 2 diabetes characterised by a small peak in hospital bed-day rate in young age group is particularly worrying. Importantly, about 40% of total bed-days in young-onset type 2 diabetes were caused by mental health disorders. Previous studies suggested that mental health disorders are bidirectionally associated with type 2 diabetes and the associations are more marked in younger than older people [27]. Although type 2 diabetes is not common in young age, the incidence of most mental health disorders peaks in adolescents and young adulthood, with more than 60% of incident cases of all types of mental health disorders occurring before the age of 25 years worldwide [28]. The mean age of presentation of type 2 diabetes in people with mental health disorders was reported to be 10 to 20 years younger than that in people without mental health disorders [29]. The mechanisms linking type 2 diabetes and mental health disorders are complex. Heightened hypothalamic–pituitary–adrenal axis and activated sympathetic nervous system in people with mental health disorders can lead to hormonal changes and insulin resistance [30]. Certain classes of psychotropic drugs, such as second-generation antipsychotic drugs, are orexigenic and can cause weight gain and metabolic decompensation [31]. Furthermore, people with comorbid diabetes and mental health disorders had high prevalence of non-adherence to drugs and self-care, associated with suboptimal glycaemic control and frequent hypoglycaemia [32]. Although guidelines recommend screening for mental health disorders in people with type 2 diabetes and vice versa [33,34], their implementation in clinical practice is suboptimal. Up to 45% of cases of depression in people with diabetes and 70% of cases of diabetes in people with severe mental health disorders were undetected [35,36]. The high rate of co-occurrence of mental health disorders and type 2 diabetes especially in young people extended previous findings in a register-based study [5] and calls for urgent awareness program among all stakeholders to take actions. Further research is needed to provide evidence for the effectiveness of proactive screening and focused interventions for mental health disorders in young people with type 2 diabetes. In this context, our group had reported 30% risk reduction in hospitalisation in patients with type 2 diabetes who received technologically enhanced structured assessment program with risk stratification, patient empowerment, and shared decision-making compared to those receiving usual care [37].

Notably, type 2 diabetes was no longer associated with increased risk of hospital bed-days for mental health disorders in older people aged >60 years at diagnosis. In this group, the multiple morbidities might have become prominent features and it remained possible that these individuals might have undiagnosed or underreporting of mental health disorders during hospitalisation [38]. There are also differences in major subtypes of mental health disorders and clinical manifestations between the young and the elderly.

Women had a lower absolute risk but greater relative risk of all-cause and most cause-specific hospital bed-days in the presence of type 2 diabetes as compared to men. This is supported by previous studies showing that women experienced more rapid deteriorations in weight, blood lipid, and blood glucose profiles during their progression from normoglycaemia to diabetes than men [39,40]. Moreover, women with diabetes were less likely to receive optimal care and had lower attainment rates for most metabolic targets than men [41]. The sex differences in associations between type 2 diabetes and certain medical conditions were age dependent, which have not been well reported. For example, the relative risk of hospital bed-days for genitourinary conditions was greater in men compared to women diagnosed below 60 years, but the direction of this sex difference reversed in older ages. This was driven by a shift in major cause of genitourinary hospitalisations from chronic kidney disease in young people to urinary tract infection in older people.

This study has several strengths, including the territory-wide coverage of the study population across the full-age spectrum, complete capture of hospitalisation records in the public sector during the full follow-up period, and comprehensive assessment of a wide range of medical conditions. This study also has several limitations. First, this study was based on the HA EMR with inherent limitations, such as miscoding, misdiagnosis, and misclassification. However, high quality and accuracy of hospital discharge codes has been well reported in administrative data, which are sufficiently robust to support their use for research purposes [42]. Second, young people without diabetes in the 18 to 39 years age group were unlikely to be representative of those in the Hong Kong general population with potential selection bias due to inclusion of individuals in need of medical care. This could lead to underestimation of relative risk in people with young-onset diabetes. These young people (aged <40 years at index date) accounted for 4% of the total study population which should have little impact on relative risk estimates in the overall population and were unlikely to bias the trend across age groups. Third, around 10% of all inpatient bed-days in Hong Kong were provided by private sector and were not captured in the HA EMR. Fourth, we only focused on medical conditions requiring hospitalisation. Mild illnesses treated in outpatient settings were not included and were out of the scope of this study. Fifth, both the clinical threshold for hospital admission and length of inpatient stay are affected by local practice and vary across settings, which may limit the generalisability to other regions. Sixth, we did not explore factors contributing to the high risk of hospital bed-days in people with type 2 diabetes and we did not provide causal inference on these associations. Last, data on some important confounders, such as socioeconomic status, were not available in our study, and therefore, we were not able to control for them.

The findings of our study highlight the need for effective management strategies to reduce the burden of hospital bed-days in people with type 2 diabetes. It is crucial for public health policymakers and healthcare providers to be aware of age- and sex-related disparities in both absolute and relative risk of hospital bed-days associated with type 2 diabetes and to provide tailored interventions and care. The substantial burden of hospital bed-days caused by mental health disorders in young people with type 2 diabetes calls for healthcare systems to allocate adequate resources and develop targeted strategies to meet their mental health needs. Integrating mental health services into diabetes care, including routine screening for mental health conditions, early intervention, and appropriate treatment and support, has the potential to improve patient outcomes and reduce hospitalisations [43,44]. Further research is warranted to investigate the underlying factors linking type 2 diabetes and increased risk of hospital bed-days and to determine whether these associations are causal or not.

In conclusion, type 2 diabetes was associated with increased risks of hospital bed-days for a wide range of medical conditions, with an excess burden of mental health disorders in people diagnosed at a young age. These associations were more pronounced in people diagnosed at a younger than older age and varied by sex. The findings of our study suggest that age and sex differences should be considered in planning preventive and therapeutic strategies for type 2 diabetes. Integrated care and effective control of risk factors with a focus on mental health disorders are urgently needed in people with young-onset type 2 diabetes to reduce both the high absolute and relative risk of hospital bed-days in this vulnerable population.

## Supporting information

S1 Checklist STROBE Statement Checklist. (DOC)

S1 Table ICD-9 codes for the selected medical conditions. (DOCX)

S2 Table Baseline characteristics of the study population at index date. (DOCX)

S3 Table Crude hospital admission rate and median inpatient bed-days for each admission in people with type 2 diabetes and controls. (DOCX)

S4 Table Age- and sex-specific hospital bed-day rate ratios associated with type 2 diabetes for broad disease categories. (DOCX)

S5 Table Sex-specific hospital bed-day rate ratios associated with type 2 diabetes for the selected medical conditions. (DOCX)

S6 Table Hospital bed-day rate ratio for 12 medical conditions that became statistically nonsignificant at *p*-value less than 0.00036 after Bonferroni correction. (DOCX)

S1 Fig Flowchart of study population selection. (DOCX)

S2 Fig Number of people admitted to hospital by the 7 broad disease categories. (DOCX)

S3 Fig Age- and sex-specific excess absolute risk of hospital bed-days for the selected medical conditions comparing people with and without type 2 diabetes. (DOCX)

S4 Fig Crude hospital bed-day rates for the selected medical conditions in people with and without type 2 diabetes. (DOCX)

S5 Fig Age- and sex-specific hospital bed-day rate ratios for the selected medical conditions comparing people with and without type 2 diabetes. (DOCX)

## Abbreviations

CI: confidence interval
EMR: electronic medical record
HA: Hospital Authority
RR: rate ratio
