# Supplementary material for: Age- and sex-specific hospital bed-day rates in people with and without type 2 diabetes: A territory-wide population-based cohort study of 1.5 million people in Hong Kong
Source: PLoS Med. 2023 Aug 4;20(8):e1004261. doi: 10.1371/journal.pmed.1004261 (PMC10403124; doi:10.1371/journal.pmed.1004261)
Supplement: S1 Table — (DOCX) [file pmed.1004261.s002.docx]

**S1 Table. ICD-9 codes for the selected medical conditions.**

| **Broad disease categories** | **Medical conditions** | **ICD-9 codes** |
| --- | --- | --- |
| Infection/parasitis | Intestinal infection | 1-9 |
| Infection/parasitis | Tuberculosis | 10-18 |
| Infection/parasitis | Septicemia | 38 |
| Infection/parasitis | Herpes zoster | 53 |
| Infection/parasitis | Viral hepatitis | 70 |
| Neoplasms | Nasopharynx | 147 |
| Neoplasms | Esophagus | 150 |
| Neoplasms | Stomach | 151 |
| Neoplasms | Colon | 153 |
| Neoplasms | Rectum | 154 |
| Neoplasms | Liver | 155 |
| Neoplasms | Gallbladder and extrahepatic bile duct | 156 |
| Neoplasms | Pancreas | 157 |
| Neoplasms | Lung cancer | 162 |
| Neoplasms | Breast | 174 |
| Neoplasms | Uterus | 179 and 182 |
| Neoplasms | Cervis | 180 |
| Neoplasms | Ovary | 183 |
| Neoplasms | Prostate | 185 |
| Neoplasms | Bladder | 188 |
| Neoplasms | Kidney | 189 |
| Neoplasms | Non-Hodgkin lymphoma | 200 and 202 |
| Neoplasms | Multiple myeloma | 203 |
| Neoplasms | Leukemia | 204-208 |
| Mental health disorders | Dementia | 290, 294.1-294.2, and 294.8 |
| Mental health disorders | Transient mental disorders | 293 |
| Mental health disorders | Schizophrenia | 295 |
| Mental health disorders | Depression | 296.2-296.3, 300.4, and 311 |
| Mental health disorders | Bipolar | 296.0-296.1 and 296.4-296.8 |
| Mental health disorders | Delusional disorders | 297 |
| Mental health disorders | Alcohol dependence syndrome | 303 |
| Mental health disorders | Adjustment reaction | 309 |
| Circulatory system | Essential hypertension | 401 |
| Circulatory system | Ischemic heart disease | 410-414 |
| Circulatory system | Conduction disorders | 426 |
| Circulatory system | Cardiac dysrhythmias | 427 |
| Circulatory system | Heart failure | 428 |
| Circulatory system | Hemorrhage stroke | 430-432 |
| Circulatory system | Ischemic stroke | 433-434 and 436 |
| Circulatory system | Aortic aneurysm and dissection | 441 |
| Circulatory system | Peripheral vascular disease | 443 |
| Respiratory system | Acute respiratory infections | 460-466 |
| Respiratory system | Pneumonia | 480-486 |
| Respiratory system | Influenza | 487 |
| Respiratory system | Chronic obstructive pulmonary disease | 490-492, 494-496 |
| Respiratory system | Asthma | 493 |
| Respiratory system | Pleurisy | 511 |
| Digestive system | Diseases of esophagus | 530 |
| Digestive system | Peptic ulcer | 531-534 |
| Digestive system | Gastritis and duodenitis | 535 |
| Digestive system | Appendicitis | 540-543 |
| Digestive system | Hernia of abdominal cavity | 550-553 |
| Digestive system | Noninfective enteritis and colitis | 555-558 |
| Digestive system | Intestinal obstruction | 560 |
| Digestive system | Diverticula of intestine | 562 |
| Digestive system | Functional digestive disorders | 564 |
| Digestive system | Peritonitis | 567 |
| Digestive system | Chronic liver disease | 571-573 |
| Digestive system | Cholelithiasis | 574 |
| Digestive system | Cholecystitis and cholangitis | 575.0-575.1, and 576.1 |
| Digestive system | Pancreatic disease | 577 |
| Digestive system | Gastrointestinal hemorrhage | 578 |
| Genitourinary system | Nephrotic syndrome | 581 |
| Genitourinary system | Acute kidney failure | 584 |
| Genitourinary system | Chronic kidney disease | 585 |
| Genitourinary system | Renal failure | 586 |
| Genitourinary system | Kidney infection | 590 |
| Genitourinary system | Hydronephrosis | 591 |
| Genitourinary system | Kidney calculus | 592 and 594 |
| Genitourinary system | Urinary tract infection | 599.0 |
| Genitourinary system | Hematuria | 599.7 |
| Genitourinary system | Hyperplasia of prostate | 600 |
