# Supplementary material for: Age- and sex-specific hospital bed-day rates in people with and without type 2 diabetes: A territory-wide population-based cohort study of 1.5 million people in Hong Kong
Source: PLoS Med. 2023 Aug 4;20(8):e1004261. doi: 10.1371/journal.pmed.1004261 (PMC10403124; doi:10.1371/journal.pmed.1004261)
Supplement: S2 Table — (DOCX) [file pmed.1004261.s003.docx]

**S2 Table. Baseline characteristics of the study population at index date.**

| **Characteristics** | **Men (N=783,000)** | | **Women (N=733,508)** | |
| --- | --- | --- | --- | --- |
|  | **People with type 2 diabetes (N=391,500)** | **Controls (N=391,500)** | **People with type 2 diabetes (N=366,754)** | **Controls (N=366,754)** |
| Mean (SD) age at index date, years | 61.9 (12.7) | 61.9 (12.7) | 63.8 (13.5) | 63.8 (13.5) |
| Age group, n (%) |  |  |  |  |
| 18-39 years | 16,452 (4.2) | 16,452 (4.2) | 14,579 (4.0) | 14,579 (4.0) |
| 40-59 years | 158,113 (40.4) | 158,114 (40.4) | 132,373 (36.1) | 132,381 (36.1) |
| 60-79 years | 185,391 (47.4) | 185,387 (47.3) | 173,835 (47.4) | 173,831 (47.4) |
| 80-99 years | 31,544 (8.1) | 31,547 (8.1) | 45,967 (12.5) | 45,963 (12.5) |
| Mean (SD) index year | 2010 (4.9) | 2010 (4.9) | 2010 (5.0) | 2010 (5.0) |
| Index year, n (%) |  |  |  |  |
| 2002-2005 | 90,404 (23.1) | 90,450 (23.1) | 97,542 (26.6) | 95,291 (26.0) |
| 2006-2009 | 84,817 (21.7) | 84,783 (21.7) | 82,036 (22.4) | 84,284 (23.0) |
| 2010-2013 | 94,481 (24.1) | 94,475 (24.1) | 84,061 (22.9) | 84,063 (22.9) |
| 2014-2018 | 121,798 (31.1) | 121,792 (31.1) | 103,115 (28.1) | 103,116 (28.1) |
