# Supplementary material for: Age- and sex-specific hospital bed-day rates in people with and without type 2 diabetes: A territory-wide population-based cohort study of 1.5 million people in Hong Kong
Source: PLoS Med. 2023 Aug 4;20(8):e1004261. doi: 10.1371/journal.pmed.1004261 (PMC10403124; doi:10.1371/journal.pmed.1004261)
Supplement: S3 Table — (DOCX) [file pmed.1004261.s004.docx]

**S3 Table. Crude hospital admission rate and median inpatient bed-days for each admission in people with type 2 diabetes and controls**

| **Causes of hospitalisation** | **Type 2 diabetes (N=758,254)** | | **Controls (N=758,254)** | |
| --- | --- | --- | --- | --- |
|  | **Crude hospitalisation rate (per 1,000 person-years)** | **Median inpatient bed-days**  **(Interquartile range)** | **Crude hospitalisation rate (per 1,000 person-years)** | **Median inpatient bed-days**  **(Interquartile range)** |
| **Men (N=783,000)** |  |  |  |  |
| Infection/parasites | 14.5 | 6 (3, 12) | 9.6 | 6 (3, 12) |
| Neoplasms | 40.3 | 5 (2, 11) | 37.4 | 5 (2, 11) |
| Mental health disorders | 8.6 | 9 (3, 27) | 7.3 | 8 (2, 25) |
| Circulatory system | 72.3 | 4 (2, 8) | 39.5 | 4 (2, 9) |
| Respiratory system | 61.2 | 5 (3, 9) | 60.5 | 4 (2, 9) |
| Digestive system | 40.2 | 3 (2, 7) | 31.9 | 3 (2, 6) |
| Genitourinary system | 42.8 | 3 (1, 6) | 22.0 | 3 (2, 6) |
| All-cause | 463.2 | 4 (2, 8) | 331.3 | 3 (2, 8) |
|  |  |  |  |  |
| **Women (N=733,508)** |  |  |  |  |
| Infection/parasites | 13.5 | 5 (3, 10) | 8.71 | 5 (2, 9) |
| Neoplasms | 30.0 | 5 (2, 11) | 25.8 | 5 (2, 11) |
| Mental health disorders | 9.5 | 9 (3, 27) | 7.1 | 7 (2, 24) |
| Circulatory system | 62.8 | 4 (2, 9) | 37.8 | 4 (2, 9) |
| Respiratory system | 40.2 | 5 (3, 9) | 37.1 | 4 (2, 8) |
| Digestive system | 32.4 | 4 (2, 7) | 24.9 | 3 (2, 7) |
| Genitourinary system | 35.7 | 4 (2, 7) | 18.0 | 4 (2, 7) |
| All-cause | 410.1 | 4 (2, 8) | 292.2 | 4 (2, 8) |
