# Supplementary material for: Age- and sex-specific hospital bed-day rates in people with and without type 2 diabetes: A territory-wide population-based cohort study of 1.5 million people in Hong Kong
Source: PLoS Med. 2023 Aug 4;20(8):e1004261. doi: 10.1371/journal.pmed.1004261 (PMC10403124; doi:10.1371/journal.pmed.1004261)
Supplement: S4 Table — (DOCX) [file pmed.1004261.s005.docx]

**S4 Table. Age and sex-specific hospital bed-day rate ratios associated with type 2 diabetes for broad disease categories.**

| **Broad disease categories** | **Age group** | **Sex** | **Hospital bed-day rate ratio (95% CI)** | **p-value** |
| --- | --- | --- | --- | --- |
| All | 18-39 years | Men | 3.54 (3.33, 3.76) | <0.001 |
| All | 40-59 years | Men | 1.96 (1.92, 2.00) | <0.001 |
| All | 60-79 years | Men | 1.52 (1.50, 1.54) | <0.001 |
| All | 80-99 years | Men | 1.36 (1.33, 1.40) | <0.001 |
| Infection/parasites | 18-39 years | Men | 3.32 (2.65, 4.17) | <0.001 |
| Infection/parasites | 40-59 years | Men | 2.73 (2.53, 2.93) | <0.001 |
| Infection/parasites | 60-79 years | Men | 1.77 (1.70, 1.86) | <0.001 |
| Infection/parasites | 80-99 years | Men | 1.49 (1.37, 1.62) | <0.001 |
| Neoplasms | 18-39 years | Men | 3.84 (2.71, 5.44) | <0.001 |
| Neoplasms | 40-59 years | Men | 1.57 (1.48, 1.67) | <0.001 |
| Neoplasms | 60-79 years | Men | 1.41 (1.35, 1.46) | <0.001 |
| Neoplasms | 80-99 years | Men | 1.34 (1.21, 1.47) | <0.001 |
| Mental health disorders | 18-39 years | Men | 2.77 (2.16, 3.56) | <0.001 |
| Mental health disorders | 40-59 years | Men | 1.19 (1.07, 1.32) | 0.0020 |
| Mental health disorders | 60-79 years | Men | 0.93 (0.86, 1.01) | 0.068 |
| Mental health disorders | 80-99 years | Men | 1.07 (0.94, 1.23) | 0.31 |
| Circulatory system | 18-39 years | Men | 6.15 (5.26, 7.18) | <0.001 |
| Circulatory system | 40-59 years | Men | 2.61 (2.51, 2.71) | <0.001 |
| Circulatory system | 60-79 years | Men | 2.00 (1.95, 2.05) | <0.001 |
| Circulatory system | 80-99 years | Men | 1.65 (1.57, 1.74) | <0.001 |
| Respiratory system | 18-39 years | Men | 3.51 (2.88, 4.28) | <0.001 |
| Respiratory system | 40-59 years | Men | 1.78 (1.68, 1.89) | <0.001 |
| Respiratory system | 60-79 years | Men | 1.19 (1.16, 1.23) | <0.001 |
| Respiratory system | 80-99 years | Men | 1.24 (1.18, 1.29) | <0.001 |
| Digestive system | 18-39 years | Men | 3.38 (2.92, 3.90) | <0.001 |
| Digestive system | 40-59 years | Men | 1.93 (1.85, 2.01) | <0.001 |
| Digestive system | 60-79 years | Men | 1.48 (1.44, 1.52) | <0.001 |
| Digestive system | 80-99 years | Men | 1.29 (1.21, 1.37) | <0.001 |
| Genitourinary system | 18-39 years | Men | 12.7 (10.6, 15.1) | <0.001 |
| Genitourinary system | 40-59 years | Men | 4.68 (4.44, 4.94) | <0.001 |
| Genitourinary system | 60-79 years | Men | 2.04 (1.97, 2.11) | <0.001 |
| Genitourinary system | 80-99 years | Men | 1.43 (1.34, 1.53) | <0.001 |
| All | 18-39 years | Women | 4.29 (4.00, 4.60) | <0.001 |
| All | 40-59 years | Women | 2.16 (2.11, 2.21) | <0.001 |
| All | 60-79 years | Women | 1.64 (1.61, 1.66) | <0.001 |
| All | 80-99 years | Women | 1.40 (1.37, 1.42) | <0.001 |
| Infection/parasites | 18-39 years | Women | 3.42 (2.60, 4.49) | <0.001 |
| Infection/parasites | 40-59 years | Women | 2.99 (2.74, 3.26) | <0.001 |
| Infection/parasites | 60-79 years | Women | 2.00 (1.91, 2.09) | <0.001 |
| Infection/parasites | 80-99 years | Women | 1.77 (1.66, 1.88) | <0.001 |
| Neoplasms | 18-39 years | Women | 3.68 (2.88, 4.69) | <0.001 |
| Neoplasms | 40-59 years | Women | 1.87 (1.76, 1.99) | <0.001 |
| Neoplasms | 60-79 years | Women | 1.54 (1.47, 1.61) | <0.001 |
| Neoplasms | 80-99 years | Women | 1.45 (1.32, 1.60) | <0.001 |
| Mental health disorders | 18-39 years | Women | 4.75 (3.69, 6.12) | <0.001 |
| Mental health disorders | 40-59 years | Women | 1.86 (1.66, 2.08) | <0.001 |
| Mental health disorders | 60-79 years | Women | 0.98 (0.91, 1.06) | 0.57 |
| Mental health disorders | 80-99 years | Women | 1.06 (0.96, 1.17) | 0.24 |
| Circulatory system | 18-39 years | Women | 5.63 (4.46, 7.12) | <0.001 |
| Circulatory system | 40-59 years | Women | 2.74 (2.59, 2.89) | <0.001 |
| Circulatory system | 60-79 years | Women | 2.03 (1.98, 2.09) | <0.001 |
| Circulatory system | 80-99 years | Women | 1.67 (1.60, 1.74) | <0.001 |
| Respiratory system | 18-39 years | Women | 4.64 (3.74, 5.75) | <0.001 |
| Respiratory system | 40-59 years | Women | 2.12 (1.97, 2.28) | <0.001 |
| Respiratory system | 60-79 years | Women | 1.38 (1.33, 1.42) | <0.001 |
| Respiratory system | 80-99 years | Women | 1.28 (1.23, 1.33) | <0.001 |
| Digestive system | 18-39 years | Women | 3.57 (2.97, 4.29) | <0.001 |
| Digestive system | 40-59 years | Women | 2.06 (1.96, 2.17) | <0.001 |
| Digestive system | 60-79 years | Women | 1.62 (1.57, 1.67) | <0.001 |
| Digestive system | 80-99 years | Women | 1.32 (1.26, 1.38) | <0.001 |
| Genitourinary system | 18-39 years | Women | 5.21 (4.54, 6.00) | <0.001 |
| Genitourinary system | 40-59 years | Women | 3.32 (3.15, 3.50) | <0.001 |
| Genitourinary system | 60-79 years | Women | 2.48 (2.39, 2.58) | <0.001 |
| Genitourinary system | 80-99 years | Women | 1.70 (1.61, 1.79) | <0.001 |
