# Supplementary material for: Age- and sex-specific hospital bed-day rates in people with and without type 2 diabetes: A territory-wide population-based cohort study of 1.5 million people in Hong Kong
Source: PLoS Med. 2023 Aug 4;20(8):e1004261. doi: 10.1371/journal.pmed.1004261 (PMC10403124; doi:10.1371/journal.pmed.1004261)
Supplement: S5 Table — (DOCX) [file pmed.1004261.s006.docx]

**S5 Table. Sex-specific hospital bed-day rate ratios associated with type 2 diabetes for the selected medical conditions.**

| **Medical conditions** | **Broad disease categories** | **Sex** | **Hospital bed-day rate ratio (95% CI)** | **p-value** |
| --- | --- | --- | --- | --- |
| Peripheral vascular disease | Circulatory system | Women | 6.09 (4.31, 8.59) | <0.001 |
| Chronic kidney disease | Genitourinary system | Men | 6.00 (5.59, 6.45) | <0.001 |
| Peripheral vascular disease | Circulatory system | Men | 5.39 (4.52, 6.44) | <0.001 |
| Pancreas | Neoplasms | Women | 5.25 (4.25, 6.50) | <0.001 |
| Kidney failure | Genitourinary system | Men | 5.12 (3.92, 6.69) | <0.001 |
| Kidney failure | Genitourinary system | Women | 5.05 (3.80, 6.72) | <0.001 |
| Nephrotic syndrome | Genitourinary system | Men | 4.85 (3.88, 6.06) | <0.001 |
| Chronic kidney disease | Genitourinary system | Women | 4.70 (4.32, 5.11) | <0.001 |
| Nephrotic syndrome | Genitourinary system | Women | 4.46 (3.27, 6.07) | <0.001 |
| Acute kidney injury | Genitourinary system | Men | 4.34 (3.93, 4.79) | <0.001 |
| Acute kidney injury | Genitourinary system | Women | 4.03 (3.63, 4.48) | <0.001 |
| Pancreas | Neoplasms | Men | 3.91 (3.22, 4.74) | <0.001 |
| Heart failure | Circulatory system | Women | 3.89 (3.73, 4.06) | <0.001 |
| Heart failure | Circulatory system | Men | 3.79 (3.61, 3.97) | <0.001 |
| Ischemic heart disease | Circulatory system | Women | 3.51 (3.37, 3.66) | <0.001 |
| Septicemia | Infection/parasitis | Women | 3.14 (2.99, 3.29) | <0.001 |
| Pancreatic disease | Digestive system | Men | 3.04 (2.70, 3.43) | <0.001 |
| Ischemic heart disease | Circulatory system | Men | 2.98 (2.88, 3.07) | <0.001 |
| Chronic liver disease | Digestive system | Women | 2.79 (2.51, 3.10) | <0.001 |
| Kidney infection | Genitourinary system | Women | 2.79 (2.43, 3.20) | <0.001 |
| Urinary tract infection | Genitourinary system | Women | 2.69 (2.60, 2.78) | <0.001 |
| Ischemic stroke | Circulatory system | Women | 2.60 (2.49, 2.72) | <0.001 |
| Peritonitis | Digestive system | Women | 2.52 (1.86, 3.42) | <0.001 |
| Septicemia | Infection/parasitis | Men | 2.47 (2.34, 2.60) | <0.001 |
| Ischemic stroke | Circulatory system | Men | 2.34 (2.24, 2.44) | <0.001 |
| Pancreatic disease | Digestive system | Women | 2.33 (2.08, 2.62) | <0.001 |
| Transient Mental health disorders | Mental health disorders | Women | 2.29 (1.61, 3.25) | <0.001 |
| Viral hepatitis | Infection/parasitis | Women | 2.26 (1.77, 2.87) | <0.001 |
| Intestinal infection | Infection/parasitis | Men | 2.25 (2.13, 2.38) | <0.001 |
| Kidney | Neoplasms | Women | 2.20 (1.59, 3.03) | <0.001 |
| Chronic liver disease | Digestive system | Men | 2.18 (2.00, 2.37) | <0.001 |
| Viral hepatitis | Infection/parasitis | Men | 2.16 (1.88, 2.49) | <0.001 |
| Non-Hodgkin lymphoma | Neoplasms | Women | 2.16 (1.70, 2.76) | <0.001 |
| Kidney infection | Genitourinary system | Men | 2.13 (1.69, 2.68) | <0.001 |
| Cholecystitis and Cholangitis | Digestive system | Men | 2.04 (1.89, 2.19) | <0.001 |
| Leukemia | Neoplasms | Men | 2.03 (1.46, 2.81) | <0.001 |
| Multiple myeloma | Neoplasms | Women | 2.03 (1.44, 2.85) | <0.001 |
| Peptic ulcer | Digestive system | Women | 2.03 (1.90, 2.16) | <0.001 |
| Intestinal infection | Infection/parasitis | Women | 2.02 (1.93, 2.11) | <0.001 |
| Tuberculosis | Infection/parasitis | Men | 2.00 (1.82, 2.19) | <0.001 |
| Tuberculosis | Infection/parasitis | Women | 1.94 (1.66, 2.27) | <0.001 |
| Urinary tract infection | Genitourinary system | Men | 1.93 (1.85, 2.02) | <0.001 |
| Conduction disorders | Circulatory system | Women | 1.92 (1.68, 2.19) | <0.001 |
| Cardiac dysrhythmias | Circulatory system | Men | 1.91 (1.83, 2.00) | <0.001 |
| Cholecystitis and Cholangitis | Digestive system | Women | 1.91 (1.77, 2.06) | <0.001 |
| Pleurisy | Respiratory system | Women | 1.90 (1.70, 2.13) | <0.001 |
| Leukemia | Neoplasms | Women | 1.90 (1.21, 2.98) | 0.0050 |
| Pleurisy | Respiratory system | Men | 1.89 (1.72, 2.07) | <0.001 |
| Kidney calculus | Genitourinary system | Women | 1.88 (1.69, 2.10) | <0.001 |
| Hydronephrosis | Genitourinary system | Men | 1.88 (1.58, 2.23) | <0.001 |
| Herpes zoster | Infection/parasitis | Men | 1.88 (1.64, 2.15) | <0.001 |
| Essential hypertension | Circulatory system | Women | 1.86 (1.80, 1.93) | <0.001 |
| Essential hypertension | Circulatory system | Men | 1.82 (1.75, 1.90) | <0.001 |
| Peritonitis | Digestive system | Men | 1.79 (1.37, 2.34) | <0.001 |
| Adjustment reaction | Mental health disorders | Men | 1.77 (1.38, 2.27) | <0.001 |
| Cardiac dysrhythmias | Circulatory system | Women | 1.76 (1.69, 1.83) | <0.001 |
| Kidney | Neoplasms | Men | 1.75 (1.39, 2.21) | <0.001 |
| Hematuria | Genitourinary system | Women | 1.74 (1.61, 1.89) | <0.001 |
| Influenza | Respiratory system | Men | 1.73 (1.63, 1.84) | <0.001 |
| Pneumonia | Respiratory system | Women | 1.73 (1.68, 1.78) | <0.001 |
| Aortic aneurysm and dissection | Circulatory system | Women | 1.72 (1.26, 2.35) | <0.001 |
| Bipolar | Mental health disorders | Men | 1.72 (1.21, 2.43) | 0.0023 |
| Influenza | Respiratory system | Women | 1.70 (1.61, 1.80) | <0.001 |
| Uterus | Neoplasms | Women | 1.70 (1.43, 2.02) | <0.001 |
| Asthma | Respiratory system | Women | 1.69 (1.53, 1.86) | <0.001 |
| Gastrointestinal hemorrhage | Digestive system | Women | 1.69 (1.59, 1.79) | <0.001 |
| Alcohol dependence syndrome | Mental health disorders | Men | 1.69 (1.28, 2.22) | <0.001 |
| Cervis | Neoplasms | Women | 1.68 (1.33, 2.13) | <0.001 |
| Conduction disorders | Circulatory system | Men | 1.67 (1.48, 1.87) | <0.001 |
| Gastritis and duodenitis | Digestive system | Men | 1.66 (1.57, 1.76) | <0.001 |
| Cholelithiasis | Digestive system | Men | 1.66 (1.56, 1.77) | <0.001 |
| Kidney calculus | Genitourinary system | Men | 1.65 (1.53, 1.77) | <0.001 |
| Liver | Neoplasms | Women | 1.64 (1.37, 1.96) | <0.001 |
| Liver | Neoplasms | Men | 1.63 (1.47, 1.79) | <0.001 |
| Colon | Neoplasms | Women | 1.62 (1.46, 1.80) | <0.001 |
| Gastrointestinal hemorrhage | Digestive system | Men | 1.62 (1.51, 1.72) | <0.001 |
| Hydronephrosis | Genitourinary system | Women | 1.62 (1.33, 1.96) | <0.001 |
| Pneumonia | Respiratory system | Men | 1.61 (1.57, 1.66) | <0.001 |
| Gallbladder | Neoplasms | Men | 1.59 (1.29, 1.97) | <0.001 |
| Non-Hodgkin lymphoma | Neoplasms | Men | 1.59 (1.30, 1.94) | <0.001 |
| Bipolar | Mental health disorders | Women | 1.59 (1.15, 2.18) | 0.0045 |
| Cholelithiasis | Digestive system | Women | 1.58 (1.48, 1.69) | <0.001 |
| Gastritis and duodenitis | Digestive system | Women | 1.58 (1.49, 1.67) | <0.001 |
| Breast | Neoplasms | Women | 1.53 (1.41, 1.67) | <0.001 |
| Noninfective enteritis and colitis | Digestive system | Men | 1.49 (1.37, 1.62) | <0.001 |
| Multiple myeloma | Neoplasms | Men | 1.49 (1.10, 2.02) | 0.011 |
| Noninfective enteritis and colitis | Digestive system | Women | 1.49 (1.39, 1.59) | <0.001 |
| Diverticula of intestine | Digestive system | Women | 1.48 (1.33, 1.65) | <0.001 |
| Hemorrhage stroke | Circulatory system | Men | 1.47 (1.37, 1.58) | <0.001 |
| Hematuria | Genitourinary system | Men | 1.47 (1.40, 1.54) | <0.001 |
| Lung | Neoplasms | Women | 1.46 (1.33, 1.61) | <0.001 |
| Herpes zoster | Infection/parasitis | Women | 1.45 (1.28, 1.65) | <0.001 |
| Peptic ulcer | Digestive system | Men | 1.44 (1.37, 1.52) | <0.001 |
| Lung | Neoplasms | Men | 1.44 (1.34, 1.55) | <0.001 |
| Prostate | Neoplasms | Men | 1.44 (1.29, 1.61) | <0.001 |
| Asthma | Respiratory system | Men | 1.43 (1.25, 1.64) | <0.001 |
| Diverticula of intestine | Digestive system | Men | 1.42 (1.27, 1.59) | <0.001 |
| Acute respiratory infections | Respiratory system | Women | 1.41 (1.35, 1.47) | <0.001 |
| Acute respiratory infections | Respiratory system | Men | 1.41 (1.35, 1.48) | <0.001 |
| Rectum | Neoplasms | Men | 1.39 (1.24, 1.56) | <0.001 |
| Diseases of esophagus | Digestive system | Women | 1.38 (1.25, 1.52) | <0.001 |
| Rectum | Neoplasms | Women | 1.38 (1.18, 1.61) | <0.001 |
| Diseases of esophagus | Digestive system | Men | 1.37 (1.25, 1.51) | <0.001 |
| Hernia of abdominal cavity | Digestive system | Women | 1.35 (1.21, 1.50) | <0.001 |
| Hemorrhage stroke | Circulatory system | Women | 1.34 (1.23, 1.46) | <0.001 |
| Transient Mental health disorders | Mental health disorders | Men | 1.33 (0.91, 1.96) | 0.14 |
| Bladder | Neoplasms | Women | 1.32 (0.92, 1.89) | 0.13 |
| Colon | Neoplasms | Men | 1.31 (1.19, 1.43) | <0.001 |
| Functional digestive disorders | Digestive system | Women | 1.30 (1.20, 1.40) | <0.001 |
| Dementia | Mental health disorders | Women | 1.29 (1.21, 1.38) | <0.001 |
| Depression | Mental health disorders | Women | 1.29 (1.12, 1.48) | <0.001 |
| Ovary | Neoplasms | Women | 1.28 (0.99, 1.65) | 0.061 |
| Aortic aneurysm and dissection | Circulatory system | Men | 1.27 (1.10, 1.45) | <0.001 |
| Stomach | Neoplasms | Women | 1.24 (1.02, 1.51) | 0.028 |
| Depression | Mental health disorders | Men | 1.24 (1.03, 1.50) | 0.026 |
| Intestinal obstruction | Digestive system | Women | 1.22 (1.09, 1.36) | <0.001 |
| Schizophrenia | Mental health disorders | Women | 1.18 (1.01, 1.39) | 0.042 |
| Dementia | Mental health disorders | Men | 1.17 (1.08, 1.26) | <0.001 |
| Adjustment reaction | Mental health disorders | Women | 1.16 (0.96, 1.41) | 0.11 |
| Functional digestive disorders | Digestive system | Men | 1.16 (1.07, 1.26) | <0.001 |
| Gallbladder | Neoplasms | Women | 1.15 (0.90, 1.48) | 0.26 |
| Stomach | Neoplasms | Men | 1.14 (0.99, 1.32) | 0.071 |
| Appendicitis | Digestive system | Men | 1.12 (0.98, 1.29) | 0.097 |
| Esophagus | Neoplasms | Women | 1.10 (0.74, 1.65) | 0.64 |
| Bladder | Neoplasms | Men | 1.10 (0.96, 1.25) | 0.16 |
| Hernia of abdominal cavity | Digestive system | Men | 1.10 (1.05, 1.14) | <0.001 |
| Hyperplasia of prostate | Genitourinary system | Men | 1.09 (1.04, 1.14) | <0.001 |
| Intestinal obstruction | Digestive system | Men | 1.04 (0.95, 1.14) | 0.41 |
| Nasopharynx | Neoplasms | Men | 1.03 (0.83, 1.29) | 0.77 |
| Chronic obstructive pulmonary disease | Respiratory system | Women | 1.01 (0.95, 1.07) | 0.81 |
| Chronic obstructive pulmonary disease | Respiratory system | Men | 0.99 (0.95, 1.04) | 0.75 |
| Delusional disorders | Mental health disorders | Men | 0.96 (0.61, 1.50) | 0.85 |
| Schizophrenia | Mental health disorders | Men | 0.93 (0.79, 1.10) | 0.40 |
| Esophagus | Neoplasms | Men | 0.91 (0.73, 1.14) | 0.43 |
| Delusional disorders | Mental health disorders | Women | 0.91 (0.64, 1.28) | 0.58 |
| Appendicitis | Digestive system | Women | 0.88 (0.77, 1.01) | 0.073 |
| Nasopharynx | Neoplasms | Women | 0.87 (0.57, 1.33) | 0.52 |
| Alcohol dependence syndrome | Mental health disorders | Women | 0.67 (0.40, 1.12) | 0.13 |
