# Supplementary material for: Age- and sex-specific hospital bed-day rates in people with and without type 2 diabetes: A territory-wide population-based cohort study of 1.5 million people in Hong Kong
Source: PLoS Med. 2023 Aug 4;20(8):e1004261. doi: 10.1371/journal.pmed.1004261 (PMC10403124; doi:10.1371/journal.pmed.1004261)
Supplement: S6 Table — (DOCX) [file pmed.1004261.s007.docx]

**S6 Table. Hospital bed-day rate ratio for 12 medical conditions that became statistically non-significant at p-value less than 0.00036 after Bonferroni correction.**

| **Medical conditions** | **Sex** | **Hospital bed-day rate ratio (95% CI)** | **p-value** |
| --- | --- | --- | --- |
| Schizophrenia | Women | 1.18 (1.01, 1.39) | 0.04226 |
| Stomach | Women | 1.24 (1.02, 1.51) | 0.02843 |
| Depression | Men | 1.24 (1.03, 1.50) | 0.02606 |
| Multiple myeloma | Men | 1.49 (1.10, 2.02) | 0.01054 |
| Leukemia | Women | 1.90 (1.21, 2.98) | 0.00498 |
| Bipolar | Women | 1.59 (1.15, 2.18) | 0.00446 |
| Bipolar | Men | 1.72 (1.21, 2.43) | 0.00228 |
| Aortic aneurysm and dissection | Men | 1.27 (1.10, 1.45) | 0.00082 |
| Aortic aneurysm and dissection | Women | 1.72 (1.26, 2.35) | 0.00068 |
| Intestinal obstruction | Women | 1.22 (1.09, 1.36) | 0.00058 |
| Functional digestive disorders | Men | 1.16 (1.07, 1.26) | 0.00045 |
| Depression | Women | 1.29 (1.12, 1.48) | 0.00044 |
