# Supplementary figures and images for: Age- and sex-specific hospital bed-day rates in people with and without type 2 diabetes: A territory-wide population-based cohort study of 1.5 million people in Hong Kong
Source: PLoS Med. 2023 Aug 4;20(8):e1004261. doi: 10.1371/journal.pmed.1004261 (PMC10403124; doi:10.1371/journal.pmed.1004261)

**S1 Fig. Flowchart of study population selection.**


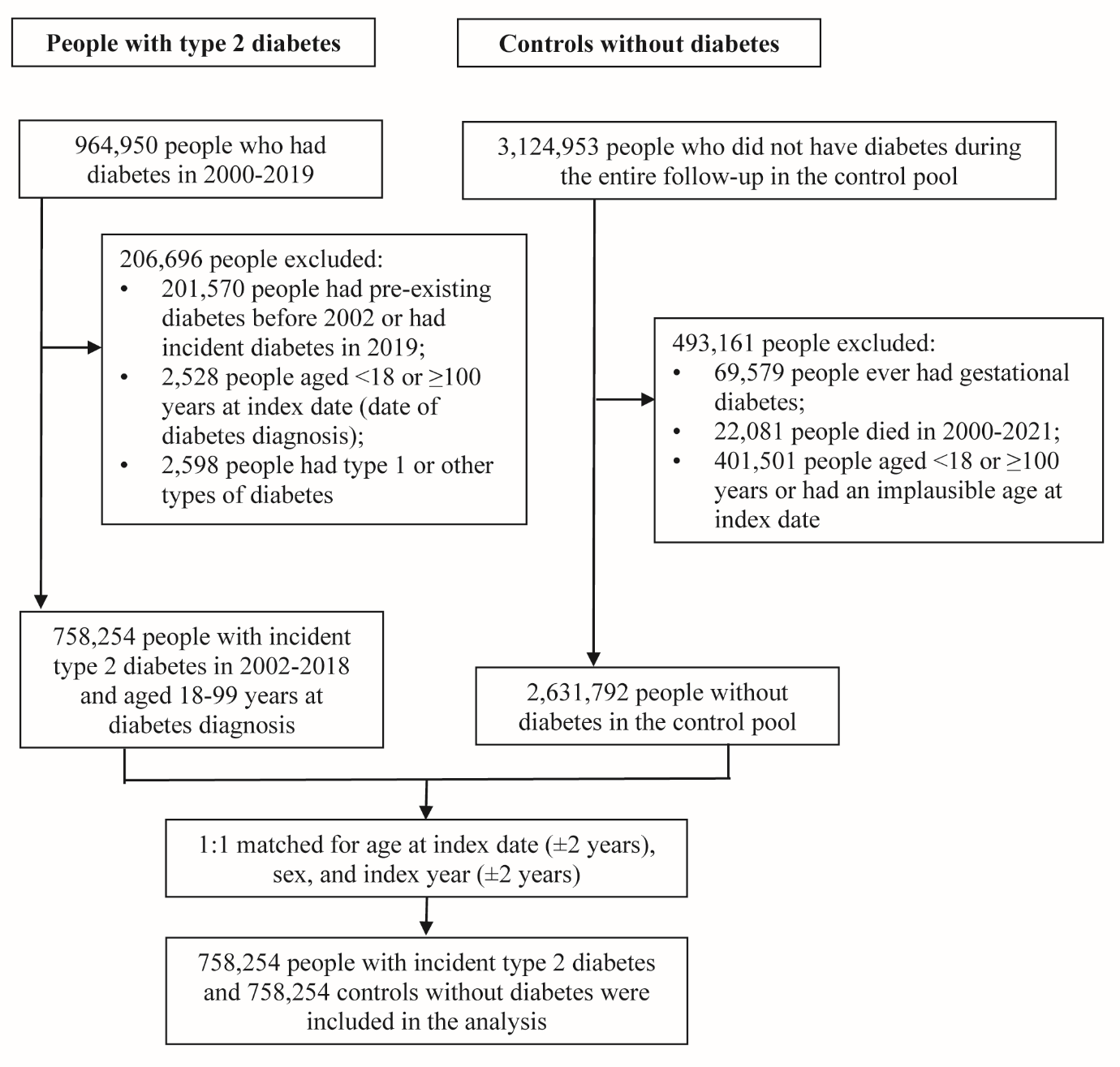

Supplement: S1 Fig — (DOCX) [file pmed.1004261.s008.docx]
