# Supplementary material for: Age- and sex-specific hospital bed-day rates in people with and without type 2 diabetes: A territory-wide population-based cohort study of 1.5 million people in Hong Kong
Source: PLoS Med. 2023 Aug 4;20(8):e1004261. doi: 10.1371/journal.pmed.1004261 (PMC10403124; doi:10.1371/journal.pmed.1004261)

**S2 Fig.** **Number of people admitted to hospital by the seven broad disease categories.** Only the top 20 leading single or combinations of broad disease categories are shown in the figures, which covered around 80% of people with hospitalisations for the seven broad disease categories. A combination of circulatory system and respiratory system indicated that people had ever been admitted to hospital due to circulatory conditions and respiratory conditions during the follow-up, regardless of temporal order. The dots/lines represent the single or combinations of broad disease categories and the bars represent the number of people admitted to the hospital.


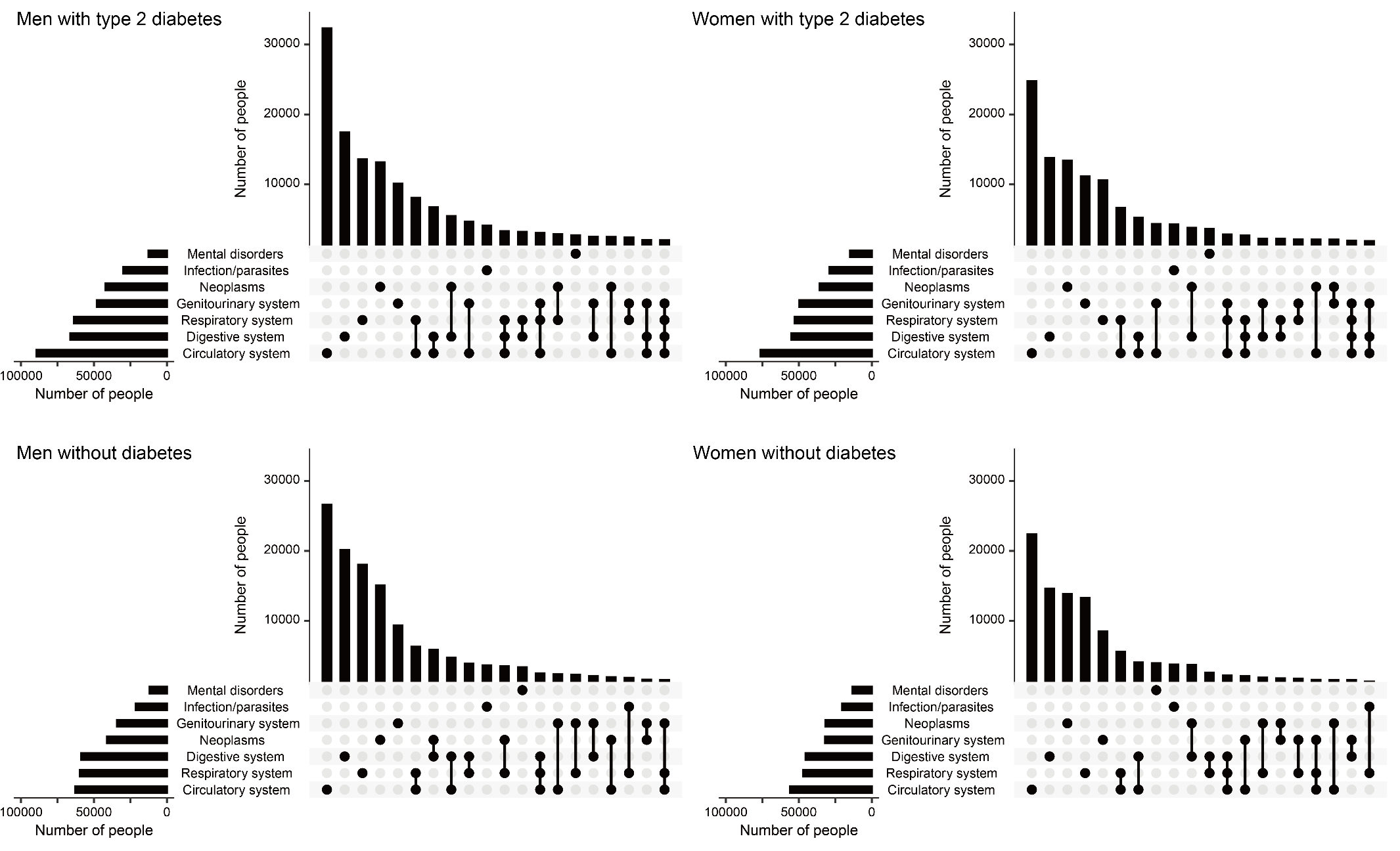

Supplement: S2 Fig — (DOCX) [file pmed.1004261.s009.docx]
