# Supplementary material for: Age- and sex-specific hospital bed-day rates in people with and without type 2 diabetes: A territory-wide population-based cohort study of 1.5 million people in Hong Kong
Source: PLoS Med. 2023 Aug 4;20(8):e1004261. doi: 10.1371/journal.pmed.1004261 (PMC10403124; doi:10.1371/journal.pmed.1004261)
Supplement: S3 Fig — (DOCX) [file pmed.1004261.s010.docx]

**S3 Fig.** **Age and sex-specific excess absolute risk of hospital bed-days for the selected medical conditions comparing people with and without type 2 diabetes.** To improve the readability of the bars in the 18-39 years age group, a magnified figure has been inserted into each panel with the y-axis limit ranging from 0 to 50.

**
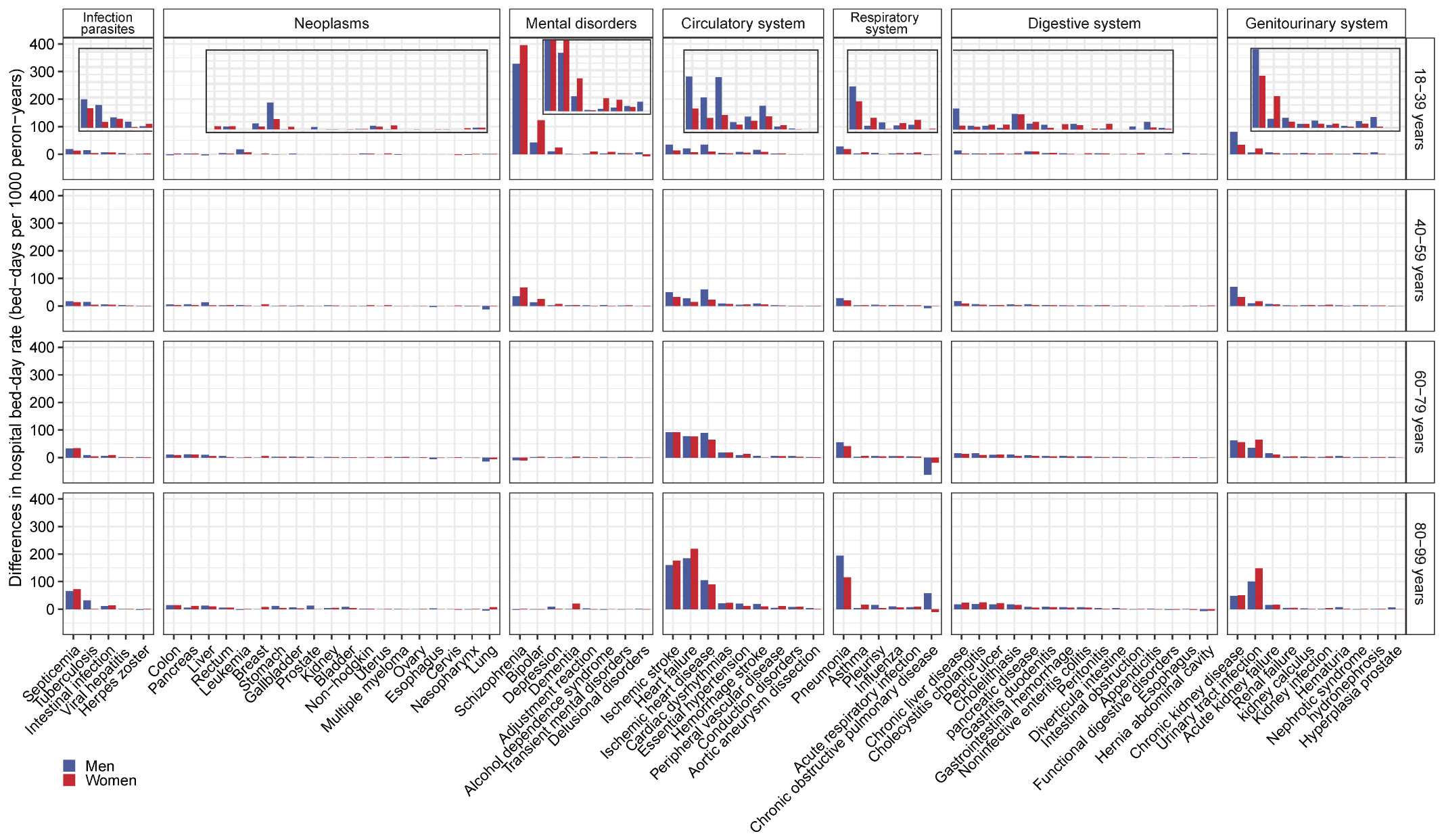
**
