# Supplementary material for: Age- and sex-specific hospital bed-day rates in people with and without type 2 diabetes: A territory-wide population-based cohort study of 1.5 million people in Hong Kong
Source: PLoS Med. 2023 Aug 4;20(8):e1004261. doi: 10.1371/journal.pmed.1004261 (PMC10403124; doi:10.1371/journal.pmed.1004261)
Supplement: S4 Fig — (DOCX) [file pmed.1004261.s011.docx]

**S4 Fig. Crude hospital bed-day rates for the selected medical conditions in people with and without type 2 diabetes.**

**
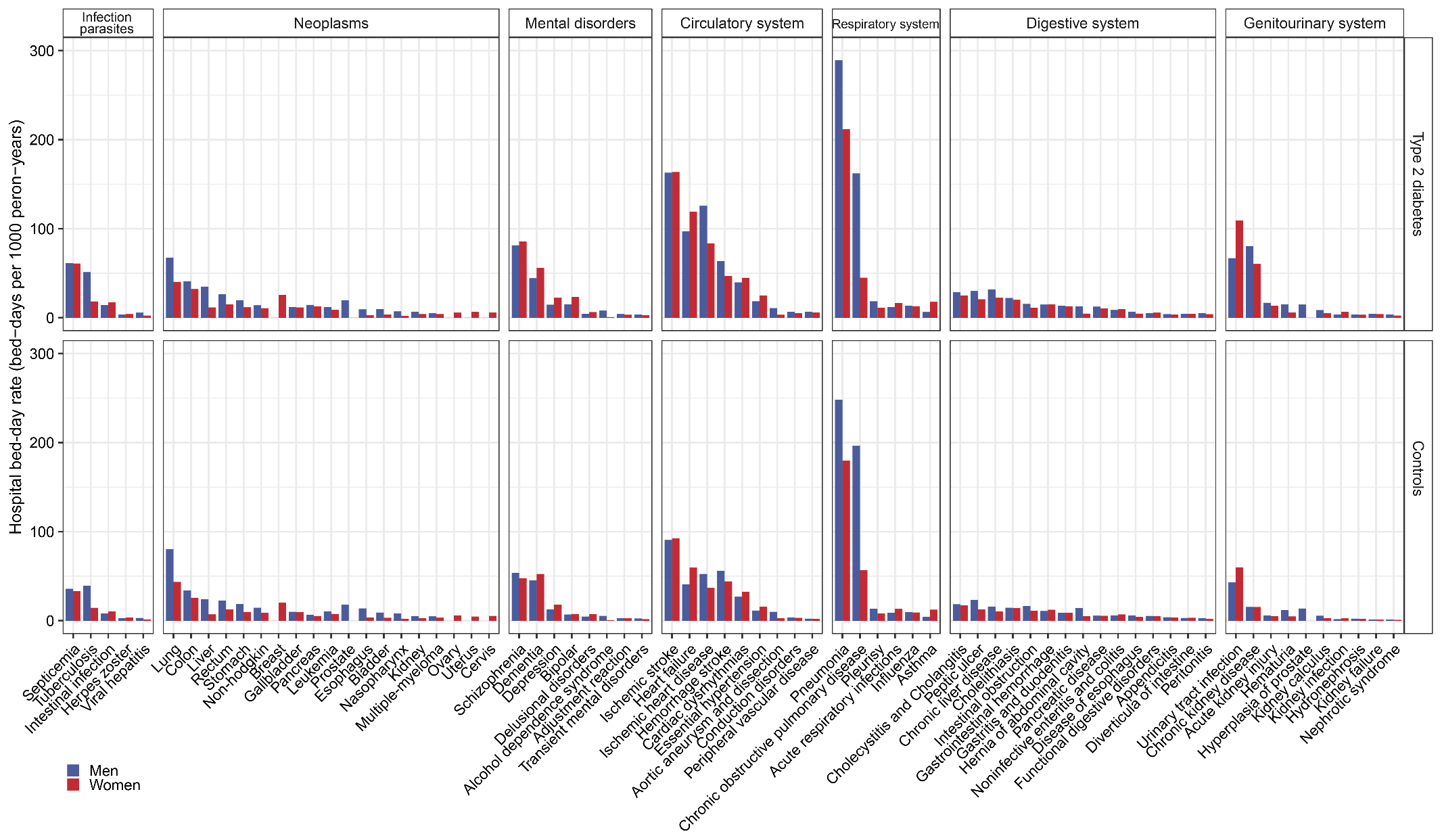
**
