# Supplementary material for: Age- and sex-specific hospital bed-day rates in people with and without type 2 diabetes: A territory-wide population-based cohort study of 1.5 million people in Hong Kong
Source: PLoS Med. 2023 Aug 4;20(8):e1004261. doi: 10.1371/journal.pmed.1004261 (PMC10403124; doi:10.1371/journal.pmed.1004261)

**S5 Fig. Age and sex-specific hospital bed-day rate ratios for the selected medical conditions comparing people with and without type 2 diabetes.** All hospital bed-day rate ratios were adjusted for age and index year. NA indicates the estimate for rate ratio was not available because there was no data or the number of events was too few to permit regression analysis.


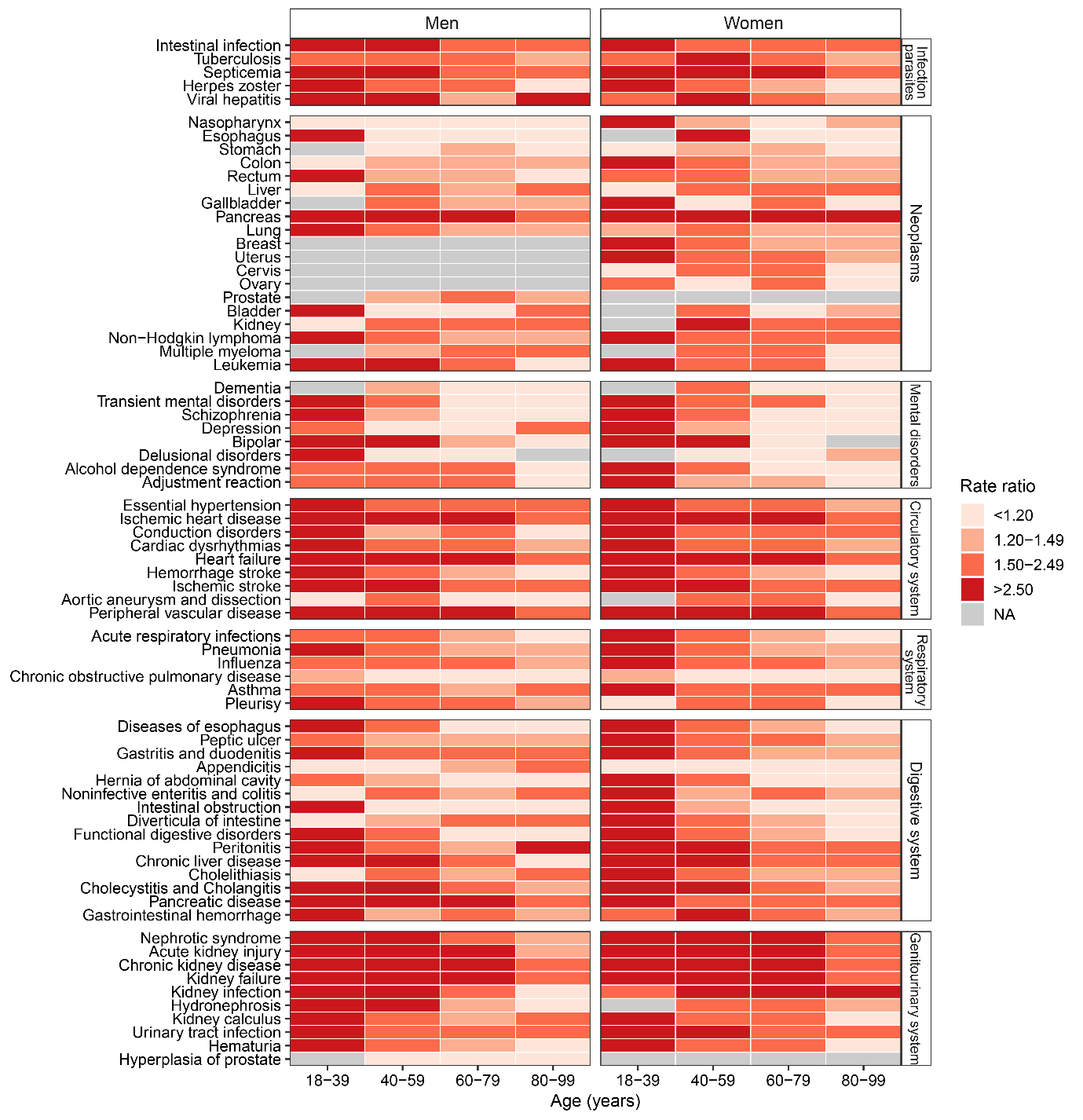

Supplement: S5 Fig — (DOCX) [file pmed.1004261.s012.docx]
